# Cavin-2 regulates the activity and stability of endothelial nitric-oxide synthase (eNOS) in angiogenesis

**DOI:** 10.1074/jbc.M117.794743

**Published:** 2017-09-14

**Authors:** Gandhi T. K. Boopathy, Madhura Kulkarni, Sze Yuan Ho, Adrian Boey, Edmond Wei Min Chua, Veluchamy A. Barathi, Tom J. Carney, Xiaomeng Wang, Wanjin Hong

**Affiliations:** From the ‡ Institute of Molecular and Cell Biology (IMCB), Agency for Science, Technology and Research (A*STAR), 61 Biopolis Drive, Proteos, Singapore,; the ¶ Lee Kong Chian School of Medicine, Nanyang Technological University, Singapore, Singapore,; the ‖ Singapore Eye Research Institute (SERI), 20 College Road, 169856 Singapore,; the ** Ophthalmology and Visual Sciences Academic Clinical Program, Duke-NUS Graduate Medical School, 8 College Rd., 169857 Singapore,; the ‡‡ Department of Ophthalmology, Yong Loo Lin School of Medicine, National University of Singapore, Singapore, and; the § SERI-IMCB Programme in Retinal Angiogenic Diseases (SIPRAD), SERI-IMCB, Singapore

**Keywords:** angiogenesis, cell biology, nitric oxide, nitric oxide synthase, signal transduction, zebrafish, eNOS

## Abstract

Angiogenesis is a highly regulated process for formation of new blood vessels from pre-existing ones. Angiogenesis is dysregulated in various pathologies, including age-related macular degeneration, arthritis, and cancer. Inhibiting pathological angiogenesis therefore represents a promising therapeutic strategy for treating these disorders, highlighting the need to study angiogenesis in more detail. To this end, identifying the genes essential for blood vessel formation and elucidating their function are crucial for a complete understanding of angiogenesis. Here, focusing on potential candidate genes for angiogenesis, we performed a morpholino-based genetic screen in zebrafish and identified Cavin-2, a membrane-bound phosphatidylserine-binding protein and critical organizer of caveolae (small microdomains in the plasma membrane), as a regulator of angiogenesis. Using endothelial cells, we show that Cavin-2 is required for *in vitro* angiogenesis and also for endothelial cell proliferation, migration, and invasion. We noted a high level of Cavin-2 expression in the neovascular tufts in the mouse model of oxygen-induced retinopathy, suggesting a role for Cavin-2 in pathogenic angiogenesis. Interestingly, we also found that Cavin-2 regulates the production of nitric oxide (NO) in endothelial cells by controlling the stability and activity of the endothelial nitric-oxide synthase (eNOS) and that Cavin-2 knockdown cells produce much less NO than WT cells. Also, mass spectrometry, flow cytometry, and electron microscopy analyses indicated that Cavin-2 is secreted in endothelial microparticles (EMPs) and is required for EMP biogenesis. Taken together, our results indicate that in addition to its function in caveolae biogenesis, Cavin-2 plays a critical role in endothelial cell maintenance and function by regulating eNOS activity.

## Introduction

Generation of blood vessels (or angiogenesis) plays a fundamental role in embryogenesis, organogenesis, and wound healing (1). Angiogenesis occurs through sequential events that are highly regulated by growth factors, cytokines, and mechanical forces (2). Abnormal or excessive formation of blood vessels due to inappropriate angiogenic signaling contributes to diseases such as age-related macular degeneration, diabetic retinopathy, atherosclerosis, rheumatoid arthritis, and cancer (3–5). These disease conditions are underscored by dysregulated angiogenic signals and therefore, there is a need to study angiogenesis in immense detail for development of better or alternative therapeutic approaches (6). Identification of genes essential for blood vessel formation and elucidating their mechanistic roles are crucial for the understanding of angiogenesis and also for the discovery of novel therapeutic targets.

In this study, we performed a morpholino-based gene knockdown screen to identify novel players in angiogenesis. We utilized zebrafish Tg(*fli1a*:EGFP)^y1^ for our initial screen as it allows direct visualization of putative vascular phenotypes through the expression of enhanced green fluorescent protein (EGFP)^3^ under the control of the *fli1a* promoter (7). From the screen, we identify that Cavin-2 (*SDPR*; serum deprivation-response protein) is required for angiogenesis in zebrafish. Cavin-2 is a membrane-bound phosphatidylserine-binding protein and a member of the Cavin family of proteins that regulate caveolae structure and function (8, 9).

Caveolae are Ω-shaped cell surface invaginations that make up to 50% of plasma membrane and serves as a major repertoire for multiple signaling molecules including endothelial nitric-oxide synthase (eNOS) in the endothelial cells (10–12). Nitric oxide (NO) produced by eNOS is crucial for systemic blood pressure, vascular remodeling, and angiogenesis (13, 14). Elevated vascular endothelial growth factor (VEGF) levels are responsible for various pathological angiogenic conditions and VEGF-induced angiogenesis can be blocked by inhibition of eNOS-mediated NO production (15, 16). Earlier, caveolin-1, one of the coat proteins of caveolae, has been shown to negatively regulate eNOS activity through its scaffolding domain and subsequently, Caveolin-1^−/−^ mice have been shown to produce significantly higher NO (17–21). The regulation of eNOS by caveolin-1 has been extensively studied but the role of other caveolae proteins on the regulation of eNOS has not been reported.

Here we demonstrate that Cavin-2 is required for cell proliferation, migration, and invasion in human umbilical vein endothelial cells (HUVECs). We show that Cavin-2 is required for *in vitro* angiogenesis in multiple endothelial cells. We find that Cavin-2 controls the production of NO by maintaining the stability and activity of eNOS in HUVECs. In addition, we find that Cavin-2 is highly secreted in endothelial microparticles (EMP) but not in exosomes and is required for EMP generation.

## Results

### Identification of genes regulating angiogenesis

**Figure 1.**
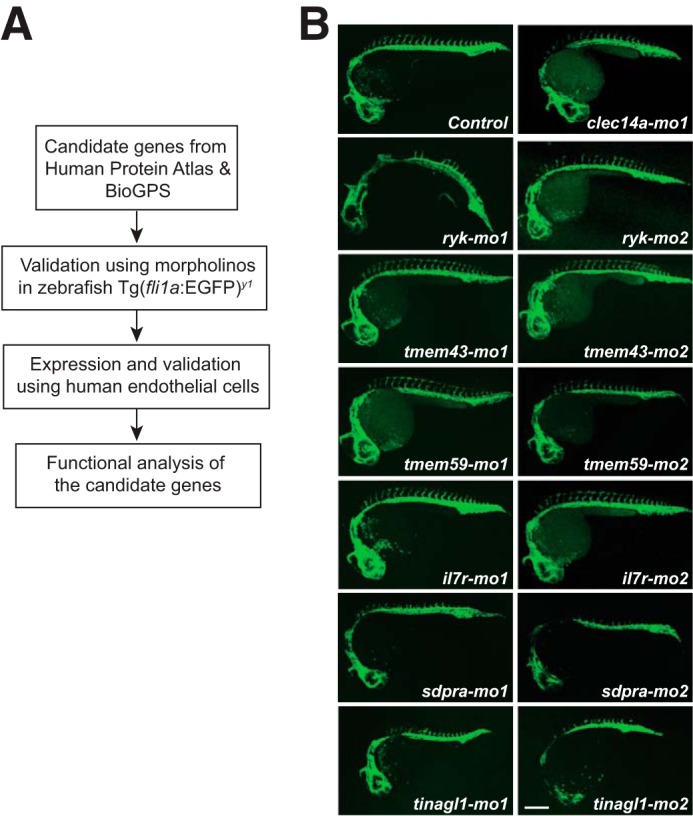
**Screen for the identification of novel angiogenesis regulator.** *A*, work flow of the screen to identify novel angiogenic regulator genes. *B*, confocal microscopy images of the microinjected candidate gene-specific morpholinos against *CLEC14A* (*clec14a-mo1*), *RYK* (*ryk-mo1* and *ryk-mo2*), *TMEM43* (*tmem43-mo1* and *tmem43-mo2*), *TMEM59* (*tmem59-mo1* and *tmem59-mo2*), *IL7R* (*il7r-mo1* and *il7r-mo2*), *SDPR* (*sdpra-mo1* and *sdpra-mo2*), *TINAGL1* (*tinagl1-mo1* and *tinagl1-mo2*) genes and control morpholino in Tg(*fli1a*:eGFP)^y1^ embryos at 24 h post-fertilization. EGFP signals from intersomitic blood vessels of Tg(*fli1a*:eGFP)^y1^ are recorded with confocal microscopy and the representative images are shown. *Scale*: 100 μm.

To identify the novel genes involved in angiogenesis, we screened a list of candidate genes from the Human Protein Atlas (HPA) and BioGPS. The “tissue atlas” in HPA has the protein expression data derived from antibody-based profiling of human proteome using immunohistochemistry (22). BioGPS is a unified source for distributed gene-annotation resources such as gene expression (23). A test scale of candidate genes were selected based on their combined protein and mRNA expression profiles restricted to blood vessels in HPA and BioGPS, respectively (Table 1). But the candidate genes weighting were based mainly on HPA as it offered a direct visualization of proteins localized in blood vessels in a variety of normal human tissues using immunohistochemistry. We utilized transgenic zebrafish Tg(*fli1a*:EGFP)^y1^ to examine their involvement in angiogenesis *in vivo*. The protein levels in zebrafish embryos can readily be reduced using morpholinos (24). We designed two independent morpholinos for each gene (one that blocks mRNA splicing and the other blocks protein translation) and tested them in zebrafish. When injected with the morpholinos against the candidate genes potentially regulating angiogenesis at the single-cell stage of zebrafish embryos, we anticipated pronounced defects in sprouting blood vessels at 24 h post-fertilization (hpf). Accordingly, our approach included a preliminarily screen of the candidate genes in zebrafish, validation of the results in human endothelial cell, and functional analysis of genes involved in angiogenesis (Fig. 1*A*).

**Table 1:** **List of candidate genes used in zebrafish screen**

No.	Gene name	Full name
1	*CLEC14A*	C-type lectin domain family 14, member A
2	*RYK*	Related to receptor tyrosine kinase
3	*TMEM43*	Transmembrane protein 43
4	*TMEM59*	Transmembrane protein 59
5	*IL7R*	Interleukin-7 receptor
6	*SDPR*	Serum deprivation response; Cavin-2
7	*TINAGL1*	Lipocalin 7

Morpholinos against *TMEM43*, *TMEM59*, and *IL7R* did not show obvious differences in intersomitic blood vessels with reference to control morpholinos (Fig. 1*B*). But the morpholinos against *CLEC14A*, *RYK*, and *TINAGL1* showed poorly connected intersomitic blood vessels. Earlier reports indicate that *CLEC14A*, *RYK*, and *TINAGL1* are suggested to be involved in angiogenesis (25–27). The *CLEC14A* gene in zebrafish is encoded by a single exon, hence we designed only protein translations blocking morpholino to target it. The complete list of morpholinos used in the zebrafish screen is available in Table 2. The evolutionally conserved role of *CLEC14A*, *RYK*, and *TINAGL1* in zebrafish suggests our approach is a reliable one.

**Table 2:** **List of morpholino sequences of candidate genes used in zebrafish screen**

No.	Gene name (human)	Oligo name	Morpholino sequences (5′-3′)
1	*CLEC14A*	clec14a-mo1	ACCATCCAGAAATCCATGTCTGCTC
2	*RYK*	ryk-mo1	GGCGACATGCTACTGGGTTTTGACG
3	*RYK*	ryk-mo2	ATCGACCAGCGCCACGGAACCTCAT
4	*TMEM43*	tmem43-mo1	TCCACCCATTGGTACATCTCCACTT
5	*TMEM43*	tmem43-mo2	ACACACACACACTGACCTCGTTGGT
6	*TMEM59*	tmem59-mo1	TCCCGTAGGACTTTATTGCACTCAT
7	*TMEM59*	tmem59-mo2	ACAAGCCGACAGTCTCGCACTCACC
8	*IL7R*	il7r-mo1	AGATAATCCACAAAAGTCCCGCCAT
9	*IL7R*	il7r-mo2	ATGTGTTAAGAAGTACCAGAGTGTT
10	*SDPR*	sdpra-mo1	CGTGAGAGGAGTCTTCACCCATCCT
11	*SDPR*	sdpra-mo2	ACTCTAAAGCATCTTCTCACCTGGA
12	*TINAGL1*	tinagl1-mo1	GCACCCAGAGCCTCAACATCGCTTA
13	*TINAGL1*	tinagl1-mo2	TTAAACTCACTGGGAGGGTAGGGAG
14		Control-mo	CCTCTTACCTCAGTTACAATTTATA

**Figure 2.**
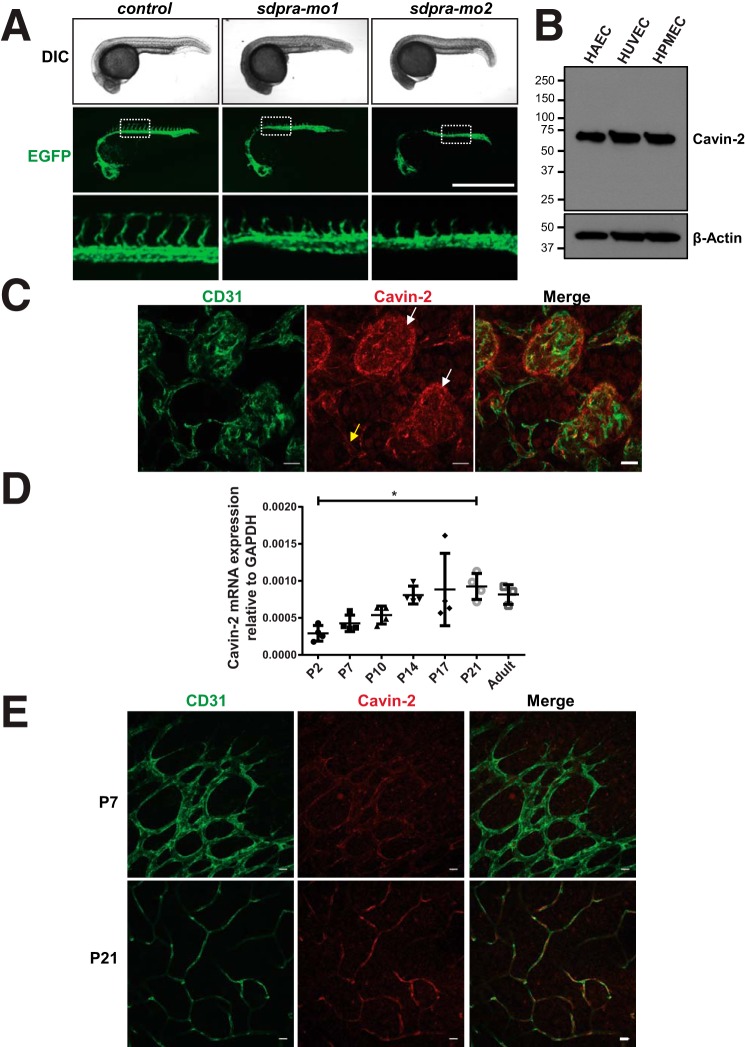
**Identification Cavin-2 as a novel angiogenesis regulator.** *A*, confocal microscopy images of the Tg(*fli1a*:eGFP)^y1^ embryos microinjected with Cavin-2 gene-specific morpholinos (*sdpra-mo1* and *sdpra-mo2*) or control morpholinos. *Top row* represents differential interference contrast (*DIC*) images, *middle row* represents EGFP signals from Tg(*fli1a*:eGFP)^y1^, and the *bottom row* represents the *zoomed inset* from the images from *middle row*. The EGFP panels shown are from the morpholino screen represented in Fig. 1*B* and represented here to show that there no major phenotypic differences between the morphants and control, and for a detailed view on the defects on intersomitic vessels of Cavin-2 morphants with respect to control morphants. *Scale*, 1 mm. *B*, immunoblots of α-Cavin-2 and α–β-actin antibodies on the WCL of HAEC, HUVEC, and HPMEC cells shows an higher expression of Cavin-2. β-Actin serves as a loading control. *C*, immunofluorescence staining of CD31 and Cavin-2 in P17 mouse retina after OIR. Cavin-2 expression is enriched at the neovascular tufts (*white arrows*), whereas low constitutive expression is associated with the surrounding non-aberrant vessels (*yellow arrow*). *Scale*, 20 μm. *D*, an increased Cavin-2 mRNA expression is associated with increased vessel density in developing mouse retina. One-way analysis of variance followed by Tukey's multiple comparisons test. Data are expressed as mean ± S.D. (*n* ≥ 3 animals); *, *p* < 0.05. *E*, the immunofluorescence staining of Cavin-2 indicates a low-constitutive and homogenous expression in retinal vasculature of P7 mouse pups and low levels in mature retinal vasculature at P21. *Scale*, 20 μm.

Additionally, our initial screen identified that the Cavin-2 orthologue, *Sdpra*, is required for angiogenesis in zebrafish (Figs. 1*B* and 2*A*). When compared with control morpholinos, injection of two independent morpholinos against Cavin-2 (both *sdpra-mo1* and *sdpra-mo2*) led to severe reduction of intersomitic blood vessels that failed to connect dorsally to form the dorsal longitudinal anastomotic vessel (Fig. 2*A, zoomed inlet*). The images of cavin-2 morphants in Fig. 2*A* were duplicated from the morpholino screen from Fig. 1*B* to show the detailed view on phenotypic and angiogenic differences between the cavin-2 morphants and control. The morpholino results indicate that Cavin-2 contributes to angiogenesis and vascular patterning, a previously unreported role. We focused further on Cavin-2 to elucidate its functional role in angiogenesis. We initially checked the protein expression levels of Cavin-2 in a panel of endothelial cells; we found that human aortic endothelial cells (HAEC), HUVEC, human pulmonary microvascular endothelial cells (HPMEC), and human retinal microvascular endothelial cells (HRMVEC) have high level of expression of Cavin-2 (Fig. 2*B* and supplemental Fig. S1).

One of the well known models to study aberrant angiogenesis in retina is the oxygen-induced retinopathy (OIR) in mice. The retinal vasculature in mice usually starts to develop around birth and is complete around 3 weeks after birth (28, 29). Exploiting this phenomenon, in OIR, 7-day-old pups are exposed to hyperoxia (70%) for 5 days (P7–P12), this exposure to high-oxygen damages the developing blood vessels in mice. After 5 days, the pups are returned to normal room air. At this point, the retina becomes hypoxic and generates a vascular overhaul response resulting in the formation of neovascular growth (tufts) that resemble the pathologic neovascularizations observed in human retinopathy of prematurity or proliferative diabetic retinopathy (28). The OIR model works efficiently as the retinal flat-mount analysis of the vasculature of the above mentioned procedure shows the formation of neovascular tufts (*top arrow*) and avascular area (*bottom arrow*) at P17 (supplemental Fig. S2*A*). Using OIR of the normal pups, we found strong staining of cavin-2 in angiogenic neovascular tufts (*white arrow*) and moderate staining of cavin-2 in normal blood vessels (*yellow arrow*) as determined based on co-staining with CD31 (Fig. 2*C*). We isolated and tested for cavin-2 mRNA expression on retina using quantitative real-time PCR (qRT-PCR) from birth until mature retinal vasculature, from P2 to P21, and adult mice. Interestingly, we find a significant increase in cavin-2 mRNA expression at P21 when compared with P2 that correlated with increased vessel density in developing mouse retina (Fig. 2*D*). Similarly, immunofluorescence staining of Cavin-2 on retinal vasculature at P7 mouse pups clearly shows a low-homogenous expression and moderate levels in mature retinal vasculature at P21 (Fig. 2*E*). The high staining of Cavin-2 in neovascular tufts and gradual increase in mRNA expression during postnatal development clearly indicate that Cavin-2 is likely to be involved in angiogenesis. To rule out the possibility of nonspecific antibody staining, we tested only anti-rabbit secondary antibody to stain neovascular tufts and found that the secondary antibody did not stain any tufts (supplemental Fig. S2*B*), indicating the Cavin-2 antibody staining is specific. High cavin-2 expression in angiogenic neovascular tufts indicates that Cavin-2 could be involved in pathogenic angiogenesis such as age-related macular degeneration and diabetic retinopathy.

### Cavin-2 is required for endothelial cell proliferation, migration, and invasion in vitro

**Figure 3.**
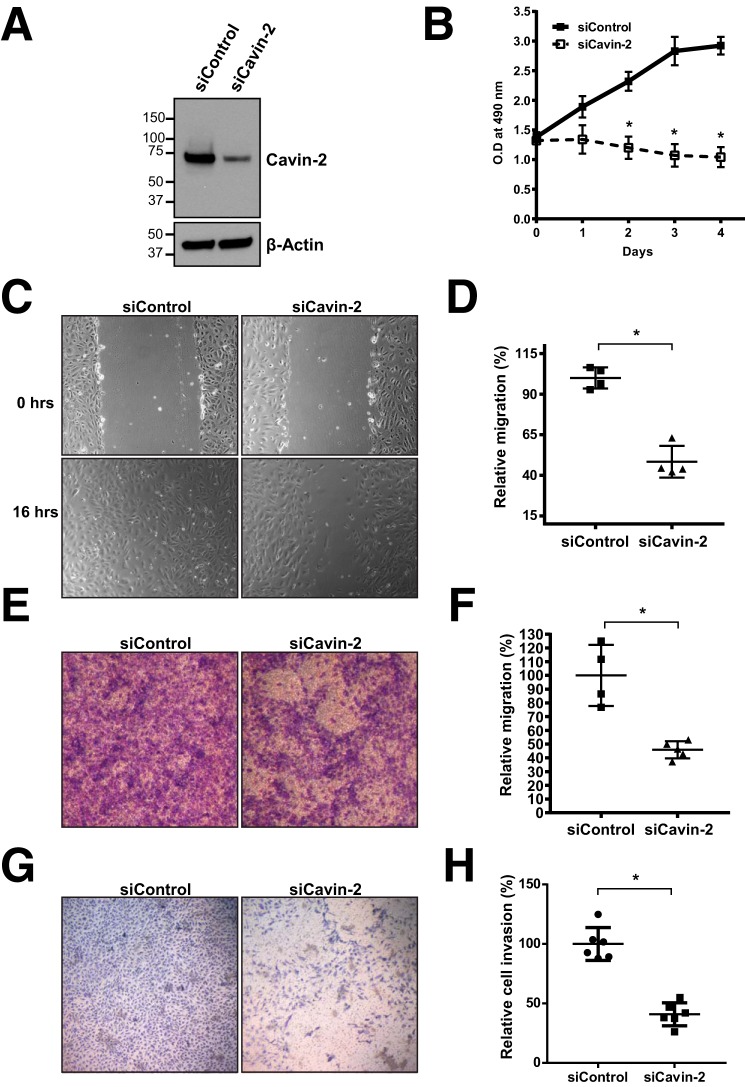
**Cavin-2 is required for cell proliferation, migration, and invasion in HUVEC cells.** *A,* Western blot analysis of the WCLs from HUVECs after knockdown of non-targeting control (*siControl*) or cavin-2 (*siCavin-2*) using siRNAs shows a severe loss of Cavin-2 expression. β-Actin serves as a loading control. *B*, siControl and siCavin-2 HUVECs were analyzed for cell proliferation at the mentioned time points using MTT assay showing a defective cell proliferation of siCavin-2 HUVECs. *C*, control and Cavin-2 knockdown HUVEC cells were analyzed for cell migration using a wound healing assay. The cells shown are pictured post-scratch at 0 and 16 h showing a defective cell migration after Cavin-2 loss. *D*, areas of the wound closure were quantified at 16 h after scratch using ImageJ. *E*, cell migration analyzed using control and Cavin-2 knockdown HUVECs using a transwell migration chamber (8-micron pore diameter) after being incubated for 12 h show a defective cell migration of sicavin-2 HUEVCs. *F*, quantification of the migrated crystal violet-stained endothelial cells after 12 h of migration using ImageJ. *G*, Matrigel invasion of control and Cavin-2 knockdown HUVECs after 48 h using a Boyden chamber assay signify that cavin-2 loss severely affects invasive potential of HUVECs. The cells were fixed in 4% paraformaldehyde and stained with crystal violet. *H*, quantification of the cells invasion through the Matrigel-coated filter was performed using ImageJ. Results in the graph presented are mean ± S.D. of three independent experiments. * indicates *p* < 0.05.

To find out the role of Cavin-2 in endothelial cells, we used a “loss-of-function” approach using siRNAs. Using a pool of specific siRNAs against Cavin-2, we could ablate up to 75% of Cavin-2 expression in HUVECs (Fig. 3*A*). To form blood vessels, endothelial cells are required to migrate from the contact inhibited state, proliferate, and invade the extracellular matrix (30). We checked whether the Cavin-2-depleted endothelial cells were able to proliferate, migrate, and invade the Matrigel. For cell proliferation assays, we used non-targeting siRNA (siControl) and Cavin-2 knockdown (siCavin-2) HUVECs. An equal number of HUVECs from each condition were plated in 96 wells and followed the cell proliferation using MTT assay for 4 days. Notably, compared with siControl cells, the cell proliferation was significantly decreased from the second day until the fourth day in siCavin-2 cells (*p* = 0.025, *p* = 0.017, and *p* = 0.001 for day 2, day 3, and day 4, respectively) (Fig. 3*B*). Assuming the importance of endothelial cell migration in VEGF-mediated angiogenesis (31), we performed two independent cell-migration assays. In the wound healing assay, siControl and siCavin-2 HUVECs were grown to confluence, scratched to create a migratory space, and the cells were allowed to migrate. Interestingly, Cavin-2-silenced HUVECs migrated significantly slower compared with siControl at 16 h (*p* = 0.007) (Fig. 3, *C* and *D*). Also, in the transwell migration assay, Cavin-2-silenced HUVECs had a significantly reduced migratory potential than control HUVECs (*p* = 0.0129) (Fig. 3, *E* and *F*). In the Matrigel invasion assay, Cavin-2 knocked down HUVECs had a significantly lesser ability to invade the basement membrane matrix than the control HUVECs (*p* = 0.0043) (Fig. 3, *G* and *H*).

**Figure 4.**
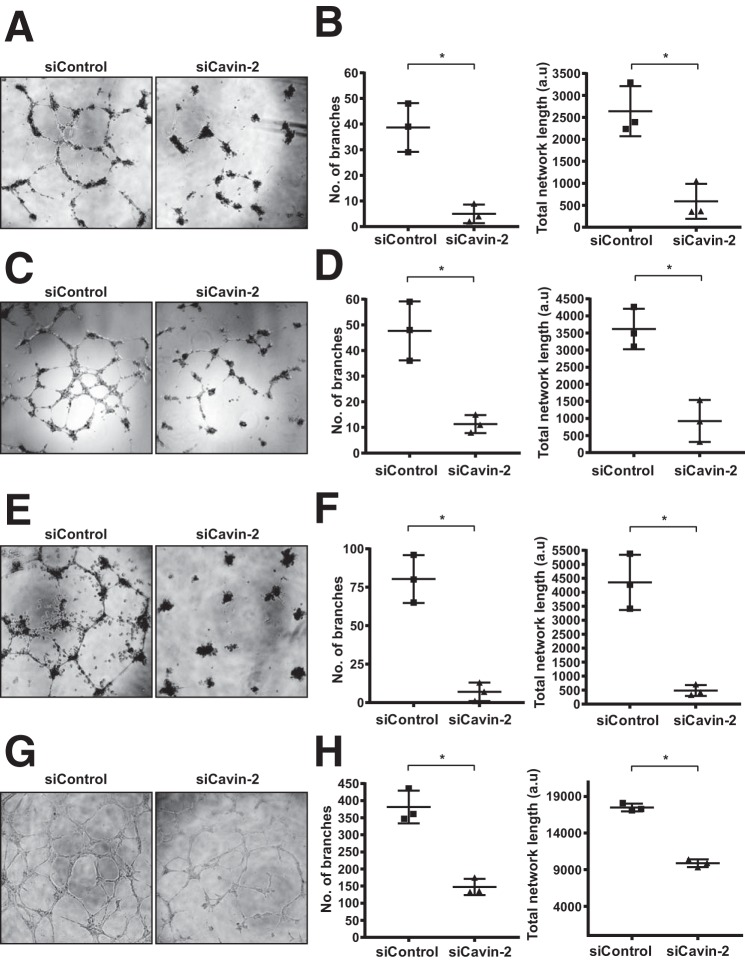
**Loss of Cavin-2 inhibits angiogenesis *in vitro* in multiple endothelial cells.** The Matrigel *in vitro* tube-formation assay was performed on control knockdown (*si-Control*) and Cavin-2 knockdown (*siCavin-2*) in the following endothelial cells: *A*, HAEC. *C*, HUVEC. *E*, HPMEC. *G*, HRMVEC. Around 5000–7000 cells with endothelial cell growth media were plated on the Matrigel basement membrane matrix and allowed to form tube-like structures and recorded using a Nikon microscope. The *graphs* represent quantitation of mean ± S.D. of the number of branches and the total network length in *in vitro* tube-formation assay of: *B*, HAEC; *D*, HUVEC; *F*, HPMEC; and *H*, HRMVEC computed using ImageJ. * indicates *p* < 0.05.

To test whether Cavin-2 is essential for angiogenesis in endothelial cells, we performed a *in vitro* tube-formation assay. Formation of capillary-like tubes by endothelial cells on a basement membrane matrix (Matrigel) in *in vitro* is a powerful method to screen for various factors that promote or inhibit angiogenesis (32). After Cavin-2 knockdown, *in vitro* tube formation in Matrigel were performed in four different types of endothelial cells. In all four endothelial cell types: HAEC (Fig. 4*A*), HUVEC (Fig. 4*C*), HPMEC (Fig. 4*E*), and HRMVEC (Fig. 4*G*), Cavin-2 ablation severely affected the tube-forming ability of endothelial cells. We then quantified the number of branches and the total network length from the *in vitro* angiogenesis assays using ImageJ. The number of branches and the endothelial tubular network were significantly reduced after silencing Cavin-2 in HAEC (Fig. 4*B, p* = 0.022 and *p* = 0.009, respectively), HUVEC (Fig. 4*D*, *p* = 0.031 and *p* = 0.025, respectively), HPMEC (Fig. 4*F, p* = 0.008 and *p* = 0.012, respectively), and HRMVEC (Fig. 4*H, p* = 0.022 and *p* = 0.006, respectively) when compared with the controls.

**Figure 5.**
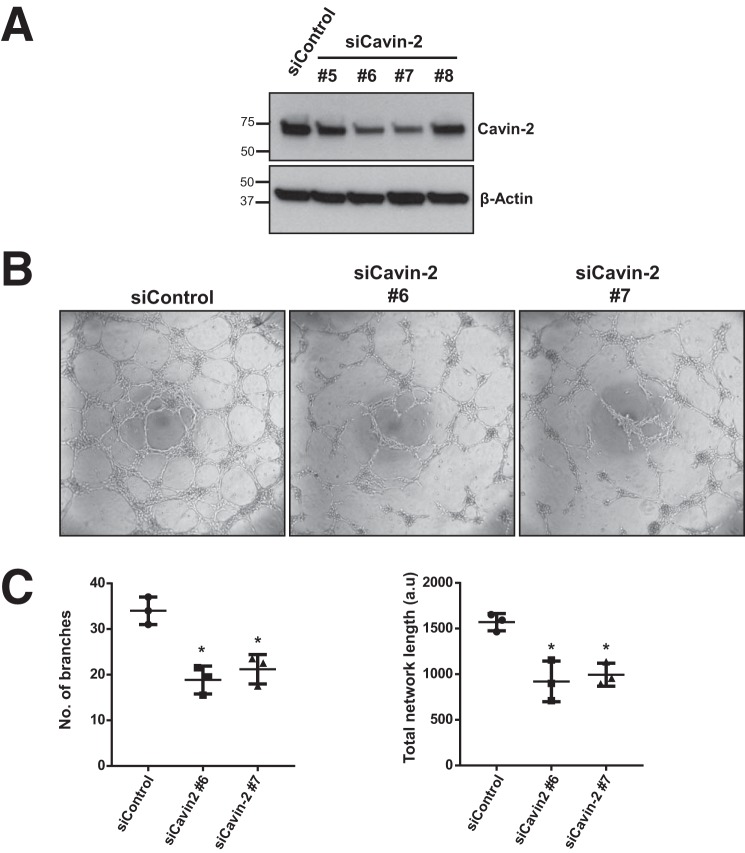
**Validation of the specificity of Cavin-2 knockdown in HUVECs.** HUVEC cells were knocked down with individual siRNAs (numbers 5, 6, 7, and 8) against Cavin-2 (*siCavin-2*) and non-targeting control (*siControl*). *A*, WCLs were analyzed in a Western blot for Cavin-2 and β-Actin. Cavin-2 protein expression was severely lost in siRNA numbers 6 and 7. β-Actin serves as a loading control. *B*, *in vitro* angiogenesis assay on control and two individual Cavin-2 siRNAs (numbers 6 and 7) in HUVECs show a defective tube formation upon loss of cavin-2. *C*, graphs represent the quantitation of mean ± S.D. of the number of branches and the total network length in HUVECs computed using ImageJ from three independent experiments. *, indicates *p* < 0.05.

To validate the effect of knockdown of cavin-2 were not due to the off-target effect of siRNAs, we deconvoluted the siRNA pool. We used individual siRNAs from the pool and tested their knockdown efficiencies in HUVECs. We find that two individual siRNAs (numbers 6 and 7) have strong knockdown efficiency than other Cavin-2 siRNAs (Fig. 5*A*). In HUVECs, we performed the *in vitro* angiogenesis assay using these two efficient siRNAs (Fig. 5*B*). Reassuringly, the two efficient siRNAs against Cavin-2 produced significantly lesser branches (*p* = 0.029 and 0.005 for numbers 6 and 7, respectively) and endothelial tubular network (*p* = 0.024 and 0.021 for numbers 6 and 7, respectively) in HUVECs (Fig. 5*C*). Two independent siRNAs specific to cavin-2 resulting in defective *in vitro* angiogenesis clearly show that the defective angiogenesis is indeed specific to the loss of cavin-2. Taken together, the data from zebrafish and the results from *in vitro* experiments on knockdown of Cavin-2 in a variety of endothelial cells strongly suggest that Cavin-2 is required for proper angiogenesis.

### Cavin-2 regulates the activity and the stability of eNOS

**Figure 6.**
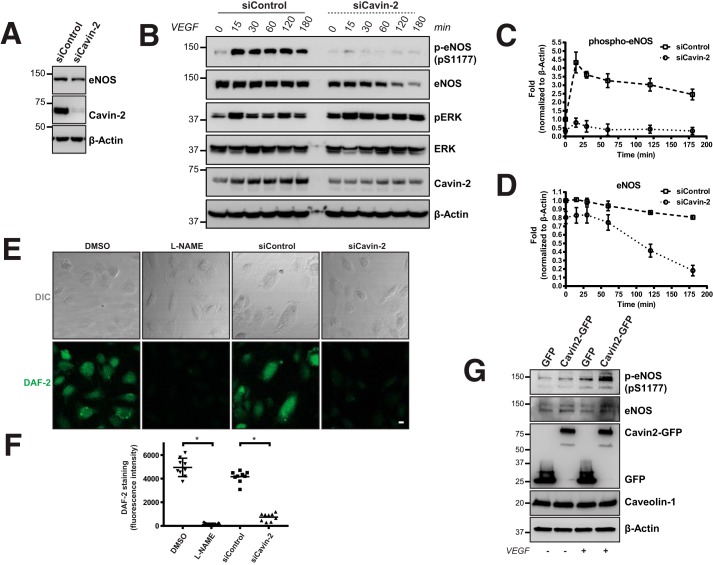
**Cavin-2 controls the activity and stability of eNOS.** *A*, WCL of control (siControl) and Cavin-2 (siCavin-2) knocked down HUVECs were separated in SDS-PAGE and immunoblotted for α-eNOS, α-Cavin-2, and α–β-Actin antibodies. *B*, siControl and siCavin-2 HUVECs were incubated for 10–12 h with endothelial cell growth medium without supplemental growth factors, stimulated with VEGF for activating eNOS, and harvested at the mentioned time points. WCL from harvested cells were immunoblotted for α-phospho-eNOS (*pS1177*), α-eNOS, α-phospho-p44/42 MAPK at Thr-202/Tyr-204 (pERK), α-p44/42 MAPK (Erk), α-Cavin-2, and α–β-Actin antibodies. β-Actin serves as a loading control. *C* and *D*, densitometry-based quantitation of phospho-eNOS (*pS1177*) (*C*) and total eNOS (*D*) normalized to β-Actin staining for the mentioned time points were performed from two independent experiments using ImageJ. *E*, live imaging of DAF-2, a cell permeable fluorescence NO indicator, upon VEGF activation on DMSO-treated, l-NAME-treated, siControl, and siCavin-2 HUVECs. The fluorescence emitted were recorded using a confocal microscope and shows a strikingly reduced DAF2 fluorescence in l-NAME-treated and siCavin-2 HUVECs. *F*, fluorescence signal intensity upon DAF-2 treatment on DMSO-treated, l-NAME-treated, siControl, and siCavin-2 HUVECs were quantified using ImageJ from at least three independent experiments. *G*, HUVECs were transfected with GFP or Cavin2-GFP plasmids, incubated for 12 h with endothelial cell growth medium without supplemental growth factors, stimulated with or without VEGF, harvested, and immunoblotted for α-phospho-eNOS (*pS1177*), α-total eNOS, α-GFP, α-Caveolin-1, and α–β-Actin antibodies. *Scale bar*, 10 μm. * indicates *p* < 0.05.

Caveolae-related proteins are very well known to play a pivotal role in multiple signaling events (33). One of the highly studied signaling pathways related to caveolae is generation of NO through eNOS (34). To elucidate the functional role of Cavin-2 in endothelial cells, we knocked down Cavin-2 in HUVECs and checked for the expression of eNOS. Interestingly, we found that the total protein levels of eNOS were decreased upon Cavin-2 knockdown in HUVECs cultured in complete endothelial cell growth medium (EGM-2) (Fig. 6*A*). Akt phosphorylation at serine 1177 activates eNOS that leads to NO production in endothelial cells (35, 36). To study whether Cavin-2 plays an important role in the stability and activity of eNOS, we followed the total eNOS and activated form of eNOS (Ser(P)-1177) during VEGF signaling. HUVECs were knocked down with Cavin-2 and control siRNAs, incubated for 10–12 h with EGM-2 media without only supplemental growth factors, treated with VEGF, and harvested at different time points up to 3 h (Fig. 6*B*). In control knockdown cells, the activity of eNOS at 15 min and later time points when compared with 0 min clearly indicate the activation of the VEGF pathway. Strikingly, we find that eNOS is not active even after multiple time points of VEGF activation in siCavin-2. This is quite interesting as none of the caveolar proteins' deficit is implicated in the loss of eNOS activity. The total protein levels of eNOS after the loss of Cavin-2 is reduced in earlier time points when compared with the control. Quite interestingly, the total eNOS levels were also strikingly declined in later time points, at around 3 h, most of the eNOS is degraded (Fig. 6*B, second blot from top*). Densitometry based quantitation of the phospho-eNOS in two independent experiments clearly show that the activity of eNOS is decreased by more than 5-fold (4.32 ± 0.608 *versus* 0.811 ± 0.268) in siCavin-2 cells at 15 min, where the activity of eNOS is at the highest in control (Fig. 6*C*). The quantification of total eNOS shows that there is a decrease in the total eNOS protein levels in the control cells, 3 h post-stimulation. There is more than a 4-fold (0.804 ± 0.015 *versus* 0.182 ± 0.061) decrease in total eNOS levels in Cavin-2 knockdown cells when compared with the control (Fig. 6*D*). These experiments clearly show that the loss of Cavin-2 significantly decreases the activity and stability of eNOS in HUVECs.

We anticipated that the loss of eNOS activity upon Cavin-2 loss should decrease the total amount of NO produced in the endothelial cells. To measure the total intracellular NO upon loss of Cavin-2 in intact cells, we used the cell-permeable fluorescence NO indicator 4,5-diaminofluorescein diacetate (DAF-2) (37). Fluorescent triazolofluorescein is formed upon reaction of NO with DAF-2 and the fluorescence yielded can be readily detectable using a fluorescence microscope. We performed DAF-2 fluorescence in control and cavin-2 knockdown HUVECs along with HUVECs treated with 1 mm *N*^ω^-nitro-l-arginine methyl ester hydrochloride (l-NAME), an inhibitor of eNOS or DMSO alone. HUVECs were treated with VEGF for 15 min, DAF-2 fluorescence staining was performed, the cells were washed with PBS and recorded live using a confocal microscope. As expected, DAF-2 fluorescence was abrogated in the reduced l-NAME-treated HUVECs than the DMSO-treated cells (*p* < 0.0001) (Fig. 6, *E* and *F*). Interestingly, Cavin-2 knockdown HUVECs exhibited a more severe loss of DAF-2 fluorescence than the control cells (*p* < 0.0001) (Fig. 6, *E* and *F*). This clearly shows that Cavin-2 is required for NO production in HUVECs.

We hypothesized that if the cavin-2 deficit causes severe loss of eNOS activity, overexpression of cavin-2 should elevate the eNOS activity in HUVECs. To test this we transfected plasmids containing cavin2-GFP or GFP in HUVECs, after 24 h we incubated the cells in EGM-2 media without supplemental growth factors for 12 h, treated with or without VEGF for 15 min, and the cell lysates were immunoblotted. Interestingly, the results show that the activity of eNOS was strongly increased upon VEGF-treated HUVECs transfected with cavin-2 than the GFP-transfected HUVECs (Fig. 6*G*). Interestingly, the loss of activity and stability of eNOS upon ablation of any caveolae-related proteins is not reported. Together, these experiments clearly show that Cavin-2 controls the activity and stability of eNOS.

### Regulation of eNOS by Cavin-2 is independent of caveolae

**Figure 7.**
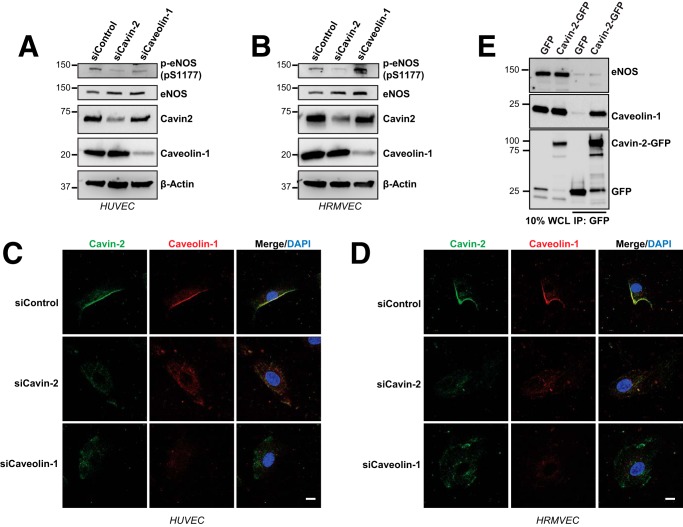
**Caveolae are not required for the regulation of eNOS by Cavin-2.** *A* and *B*, HUVEC (*A*) and HRMVEC (*B*) were knocked down with control (*siControl*), Cavin-2 (*siCavin-2*), and Caveolin-1 (*siCaveolin-1*) using specific siRNAs. Cells were incubated in endothelial cell growth medium without supplemental growth factors for 12 h, stimulated with VEGF, harvested after 15 min, and immunoblotted for α-phospho-eNOS (*pS1177*), α-total eNOS, α-Cavin2, α-Caveolin-1, and α–β-Actin antibodies. *C* and *D*, immunofluorescence analyses of Cavin-2 and Caveolin-1 on HUVECs (*C*) and HRMVECs (*D*) after siControl, siCavin-2, and siCaveolin-1. Continuous staining of caveolin-1 at the plasma membrane is a characteristic of caveolae formation and the DAPI stain represents nuclei. *E*, ectopic expression and immunoprecipitation (*IP*) of Cavin-2 in HPMEC-ST1.6R cells. Cavin-2-GFP or GFP plasmid were expressed in HPMEC-ST1.6R and the WCL were used for IP. Both, WCL and IP were immunoblotted for antibodies against eNOS, Caveolin-1, and GFP. *Scale bar*, 10 μm.

Because Cavin-2 is a caveolae-resident protein, we were inquisitive whether caveolae is required for the regulation of eNOS by Cavin-2. To test this, we ablated Cavin-2 and Caveolin-1, tested for the activity of eNOS, and examined the caveolae structure in two independent endothelial cells (HUVECs and HRMVECs). After the knockdown, endothelial cells were incubated with EGM-2 media without growth factors and treated with VEGF for 15 min to activate eNOS. The results show that loss of cavin-2 clearly reduces the activity of eNOS in both HUVEC and HRMVEC than the caveolin-1 loss (Fig. 7, *A* and *B*). Caveolin-1, a negative regulator of eNOS activity is also the principal structural component of caveolae and its ablation is known to abolish caveolae formation (19). The continuous immunofluorescence staining of caveolin-1 at the plasma membrane is a characteristic of caveolae formation (Fig. 7, *C* and *D, top row*). In both HUVEC and HRMVEC, caveolae are lost in cavin-2 knockdown cells similar to siCaveolin-1 cells (Fig. 7, *C* and *D, middle* and *bottom rows*) but the total Caveolin-1 levels remain unchanged in HUVEC and HRMVEC (Fig. 7, *A* and *B*). However, the caveolin-1 loss has been implicated to increase eNOS activity in mice (38, 39). Together, the eNOS activity assay with mice studies on caveolin-1^−/−^ and the immunofluorescence data of caveolae on siCavin-2 and siCaveolin-1 in endothelial cells show that the caveolae are not required for the regulation of eNOS by Cavin-2. Also, in our earlier experiment (Fig. 6*G*), overexpression of cavin-2 in HUVECs showed an increase in eNOS activity, however, in the same experiment the total caveolin-1 levels were not altered when compared with the control, suggesting unaltered levels of caveolae in these cells further confirms that cavin-2 regulates eNOS independent of caveolae.

Caveolin-1 is known to be directly interacting with eNOS to negatively regulate the latter's activity (21). We hypothesized that Cavin-2 may interact and regulate the activity of eNOS. To validate this, we transfected Cavin2-GFP or GFP alone in the HPMEC-ST1.6R cell line, an endothelial cell line derived from human pulmonary microvascular endothelial cells (40). We did immunoprecipitation of Cavin-2 using GFP-antibody at the basal state; interestingly, we could not detect the interaction of eNOS with Cavin-2 (Fig. 7*E*). Fig. 7*E* (*bottom*) clearly shows that we could efficiently pull Cavin-2 and GFP. As a well known interaction partner of Cavin-2, we could detect the interaction of Cavin-2 with Caveolin-1 in our immunoprecipitation (Fig. 7*E*, *middle panel*) as a positive control (9). Together these results suggest that Cavin-2 is not in a complex with eNOS but regulates through an unknown mechanism that remains to be elucidated.

**Figure 8.**
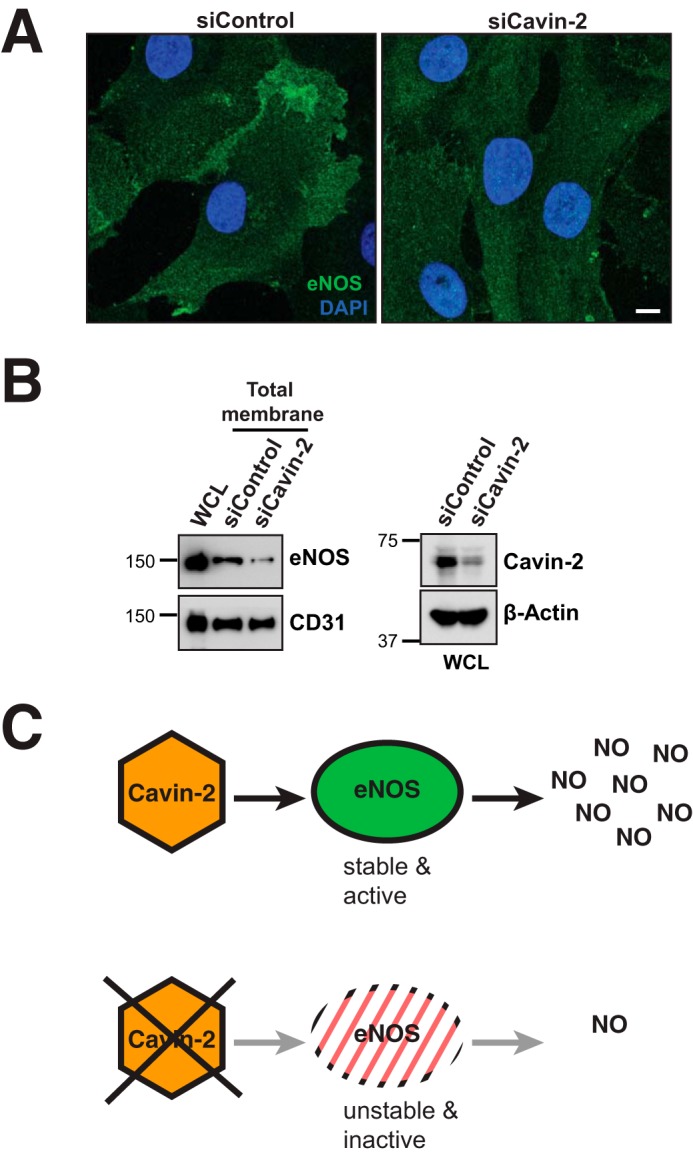
**Cavin-2 controls the cellular localization of eNOS.** *A*, immunofluorescence of α-eNOS antibody on control (*siControl*) and Cavin-2 (*siCavin-2*) knocked down HUVECs. DAPI stain represents nuclei. *B*, immunoblots of eNOS and CD31 on total membrane isolated from an equal number of siControl and siCavin-2 HUVECs. Total membranes were resolved along with WCL of untreated HUVECs on SDS-PAGE and transferred to nitrocellulose membrane. On the *right side*, immunoblots of WCL of siControl and siCavin-2 HUVECs with α-Cavin-2 and α–β-Actin antibodies. β-Actin serves as a loading control. *C*, scheme of the regulation of NO production by Cavin-2 in endothelial cells. The presence of Cavin-2 positively helps in NO production by stabilizing and activating eNOS. The loss of Cavin-2 adversely effects NO production by destabilizing and inactivating eNOS. *Scale bar*, 10 μm.

Earlier reports suggested that the proximity of eNOS at the plasma membrane is required for its proper function (41). Therefore, we decided to investigate the localization of eNOS upon ablation of Cavin-2. We did immunofluorescence on control and cavin-2 knockdown HUVEC cells with eNOS antibody and recorded the samples using a confocal microscope. The results clearly show that eNOS is considerably localized on the plasma membrane in control knockdown cells but the plasma membrane localization of eNOS is considerably lost upon cavin-2 loss (Fig. 8*A*). To validate the eNOS antibody for its specificity to recognize eNOS, we knocked down eNOS using specific siRNAs (sieNOS) or non-targeting siRNA (siControl) and checked the immunofluorescence staining of eNOS in HUVECs. Supplemental Fig. S3*A* clearly shows that upon knockdown, total eNOS protein levels were strikingly lost and similarly immunofluorescent staining of eNOS in sieNOS was also lost, suggesting that the eNOS antibody is quite specific (supplemental Fig. S3*B*). To further validate the loss of eNOS at the plasma membrane upon ablation of cavin-2, we isolated the total membrane from a similar number of control and Cavin-2 knockdown cells and immunoblotted for eNOS and CD31 (Fig. 8*B*). The CD31 levels at the plasma membrane in both the control and cavin-2 knockdown show an equal loading of membranes but interestingly, we find that eNOS levels were strongly reduced in cavin-2 knockdown cells. The immunoblot with cavin-2 and β-Actin show the loss of cavin-2 and equal loading in WCL, respectively (Fig. 8*B, right*). These two independent experiments clearly show that eNOS is lost at the plasma membrane upon Cavin-2 knockdown, suggesting that cavin-2 may play a role in membrane localization of eNOS. Taken together, all our results show that cavin-2 regulates the activity and stability of eNOS to produce NO in endothelial cells and the loss of cavin-2 inactivates and destabilizes eNOS to impair NO production (Fig. 8*C*).

### Cavin-2 is secreted in endothelial microparticles

**Figure 9.**
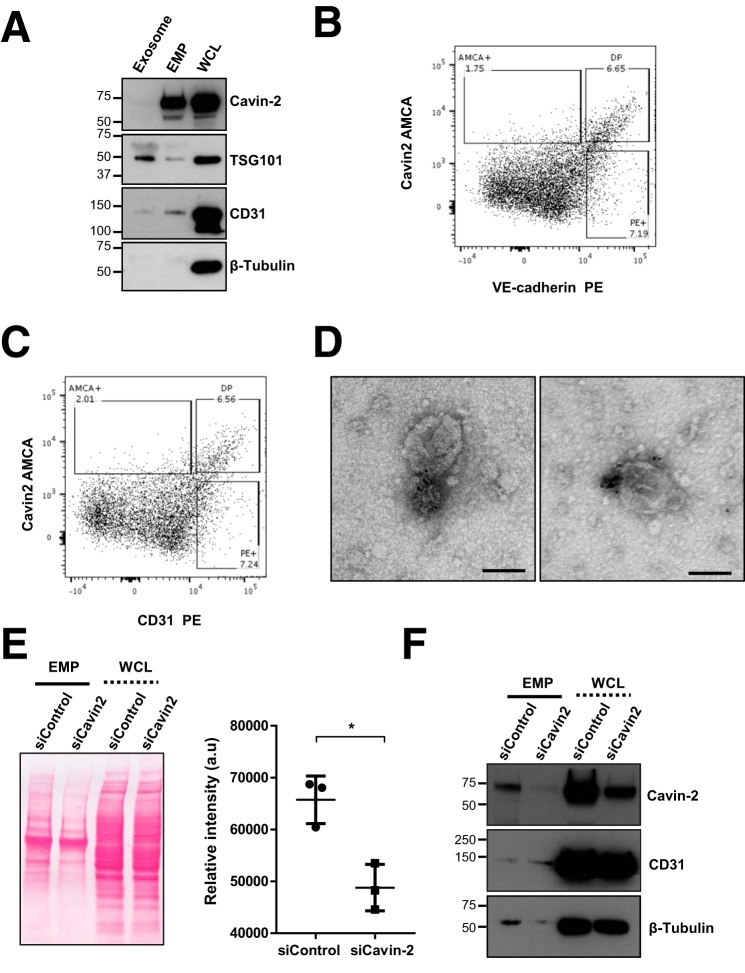
**Cavin-2 is secreted in EMP.** *A*, conditioned media from HUVECs were collected, exosomes and EMP were isolated, separated along with WCL in SDS-PAGE, and immunoblotted to test for the presence of Cavin-2 in exosomes (*TSG101*), microparticles (*CD31*), and cytosol (β-*Tubulin*). Isolated EMPs were sorted for 0.1 to 1-μm sized particles using nano blank polystyrene beads as reference in flow cytometry. EMPs were analyzed for co-staining of Cavin-2 with endothelial cell surface markers, *B* and *C*, VE-cadherin (*B*) and CD31 (*C*). α-Cavin-2 antibody is conjugated with AMCA, both α-VE-cadherin and α-CD31 antibodies were conjugated using PE. *D*, localization of Cavin-2 on EMPs with α-Cavin-2 antibody followed by goat α-rabbit secondary antibody coupled with 10-nm gold and analyzed using transmission electron microscope. Two representative images from independent experiments were shown. *Scale bar*, 100 nm. *E*, EMPs were isolated and concentrated from an equal number of control (siControl) and Cavin-2 (siCavin-2) knockdown HUVECs. Isolated EMPs were separated along with WCL of siControl and siCavin-2 cells in SDS-PAGE, transferred to nitrocellulose membrane, and stained using 0.1% Ponceau S staining reagent. The total protein abundance stained using Ponceau S of EMPs secreted from siControl and siCavin-2 HUVEC were quantified using ImageJ, the signal intensities were plotted from three independent experiments (*a.u.,* arbitrary units). *F*, immunoblotting of EMPs and WCLs from siControl and siCavin-2 HUVECs with α-Cavin-2, α-CD31, and α–β-Tubulin antibodies.

Secreted proteins from endothelial cells often stimulate endothelial cell function through auto and paracrine signaling (42). Therefore, we performed a mass spectrometry analysis of secreted proteins (secretome) of endothelial cells to identify the prominent secreted proteins. Interestingly, in our mass spectrometry screen, we found that Cavin-2 is highly secreted from HUVECs (Table 3; iBAQ value, 8287400). Endothelial cells communicate by releasing membrane vesicles in the form of exosomes and microparticles. To find exactly how Cavin-2 is secreted from endothelial cells, we isolated both exosomes and EMP (or microparticles). EMPs are vesicle-like structures, quite irregular and intact with the size range 0.1–1.0 μm, released from the surface of endothelial cells through plasma membrane blebbing in the healthy and pathological states (43), whereas exosomes are less than 100 nm in diameter, and produced by multivesicular bodies. Immunoblot of exosomes, EMPs, and the WCL from HUVECs clearly show that cavin-2 is highly secreted in EMPs and not in exosomes (Fig. 9*A*). Immunoblots probed with exosome and EMP markers TSG101 and CD31, respectively, clearly show distinction between exosome and EMP isolates. EMPs can readily be sorted based on its ability to bind to Annexin V (44). To validate that our EMPs are indeed distinct from cell debris and apoptotic bodies, EMPs were isolated and subjected to Annexin V staining. The EMP samples were incubated with Annexin V-Fluos labeling reagent in the dark at room temperature and immediately analyzed using flow cytometry. As an internal reference for the size and complexity, we used polystyrene standard size beads to sort EMPs between 0.1 and 1.0 μm diameters. The size smaller than 1.0 μm and the shift of fluorescence in the histogram after specific labeling of Annexin V indicates that these particles were indeed EMPs (supplemental Fig. S4). To further confirm that cavin-2 is in the EMPs, we conjugated aminomethylcoumarin acetate (AMCA) with Cavin-2 antibody and R-phycoerythrin (PE) with vascular endothelial (VE)-cadherin or CD31 antibodies. We isolated and stained EMPs with Cavin-2 and co-stained endothelial cell surface markers, VE-cadherin and CD31. We found that EMPs were able to co-stain cavin-2 with both VE-cadherin and CD31, respectively (Fig. 9, *B* and *C*). The isolated EMPs were also analyzed using transmission electron microscopy for the presence of cavin-2. Using immunogold labeling with α-cavin-2 antibody followed by goat anti-rabbit secondary antibody coupled with 10 nm gold, we identified cavin-2 on the surface of microparticles (Fig. 9*D*). Together, these experiments clearly show that Cavin-2 is highly secreted from endothelial cells and specifically localized in EMPs.

**Table 3:**
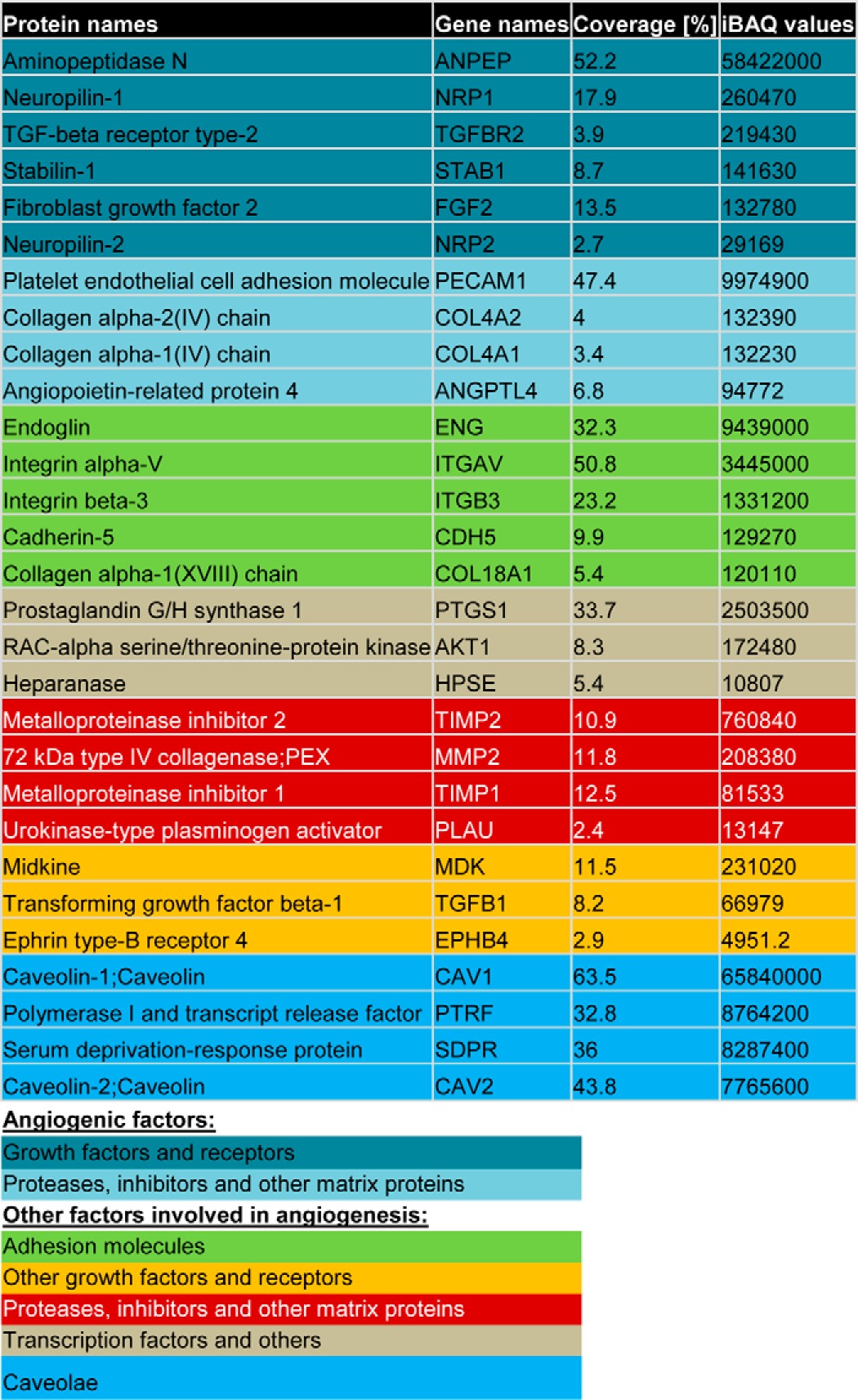
**List of angiogenic proteins identified in mass-spectometry of EMPs**

Because we detected a large quantity of cavin-2 in EMPs (Fig. 9*A*) and as earlier reports pointed to the role of lipid microdomains in the generation of microparticles, we examined whether cavin-2 has a role in the secretion of EMPs from endothelial cells (45). To test this, we knocked down Cavin-2 in HUVECs; equal numbers of cells with either siControl or siCavin-2 were plated and the EMPs secreted from these cells were isolated and examined for the overall protein content using Ponceau S staining. Clearly, a substantial amount of proteins were lost in EMPs secreted from Cavin-2 knockdown cells than the control cells (*p* = 0.01) (Fig. 9*E*). Immunoblots on the Ponceau S-stained membrane clearly shows that Cavin-2 expression is reduced after knockdown, whereas CD31 and β-Tubulin levels are similar in both siControl and siCavin-2 (Fig. 9*F*). This experiment clearly suggests Cavin-2 plays a key role in secretion of EMPs in HUVECs.

## Discussion

In this study, we identify cavin-2 as a regulator of angiogenesis based on an approach using candidate proteins identified in the Human Protein Atlas and functionally tested in zebrafish. We demonstrate that cavin-2 is required for blood vessel formation in zebrafish and controls the proliferation, migration, and invasive capabilities of endothelial cells *in vitro*. We show that cavin-2 is highly expressed in neovascular tufts in the OIR mouse model, show a significant increase in expression correlated with vessel density in developing mouse retina and required for tube formation in *in vitro* angiogenesis assays of HAEC, HUVEC, HPMEC, and HRMVEC endothelial cells. The crucial role rendered by Cavin proteins in the biogenesis and stabilization of caveolae is well studied (46). Interestingly, the physiological functions of Cavins in cell signaling are not well studied. Although Cavin-2^−/−^ mice have been reported, the physiological role of Cavin-2 in endothelial cell maintenance and function was not elucidated. Here we focused on studying the role of Cavin-2 in angiogenesis. We interestingly found that Cavin-2 regulates the functional activity and stability of eNOS in endothelial cells and controls NO production. NO produced from endothelial cells play a key role in vasodilation, inflammation, and oxidative stress (47). Reduced endothelial NO production has been associated with multiple pathogenesis including vascular disorders such as atherosclerosis and pulmonary hypertension (48, 49). Therefore, maintaining the activity and stability of eNOS becomes an important liability for endothelial cells. Most of the cellular components essential for appropriate function of eNOS are localized in caveolae or membrane microdomains (41). Acylation, both myristoylation and palmitoylation, is required for targeting eNOS to caveolae and optimal activity of eNOS (10, 41, 50, 51). The loss of membrane localization of eNOS upon Cavin-2 ablation clearly indicates that Cavin-2 is indeed required for the appropriate localization of eNOS at the caveolae and ultimately regulates the activity in endothelial cells. Earlier reports have suggested the regulation of eNOS at the transcriptional, posttranscriptional, and posttranslational levels (52, 53). At the protein level, tumor necrosis factor α (TNFα)-mediated activation of protein kinase C-ζ negatively regulates eNOS protein levels (54). Regulation of stability of eNOS is not yet reported for caveolar proteins (caveolins and cavins), in this study, upon ablation of Cavin-2, we find a decline in eNOS protein levels at the basal state and the total eNOS levels were gradually reduced to strikingly low levels at 3 h during VEGF signaling.

Interestingly, earlier reports show that similar to other caveolar genes such as Caveolin 1^−/−^ and Cavin 1^−/−^, Cavin-2^−/−^ mice were viable and fertile (20, 55). Cavin 2^−/−^ mice had perturbed lung endothelial morphology, endothelial barrier function, and loss in endothelial caveolae in lung and adipose tissues (56). Also, Hansel *et al.* (56) showed that Cavin-3 was up-regulated in a variety of tissues in Cavin 2^−/−^ mice, so we speculate that Cavin-3 may compensate the function of Cavin-2 in Cavin 2^−/−^ mice. Investigating NO production in the Cavin-2 and Cavin-3 double knock-out mice will clarify any duplicate function of Cavin-2 and Cavin-3.

EMPs are cell membrane-shed complex vesicular biological effectors that are capable of mediating vascular physiology and function (57). EMPs are generated from activated or dysfunctional endothelial cells by a variety of stimuli (58, 59). Formerly, activation of Calpain, a calcium-dependent cysteine protease, has been shown to promote membrane blebbing through degradation of cytoskeletal proteins (60). However, cellular events leading to the membrane changes for the EMPs generation are not well defined (57). We, in the current study, and others have identified a large number of lipid microdomain-related proteins (both lipid rafts and caveolae) in microparticles (61, 62). Also, lipid-rich microdomains have been associated with the formation of microparticles from a variety of cell types (45). Disruption of lipid microdomains using methyl-β-cyclodextrin or nystatin has been shown to impair the generation of microparticles in endothelial cells (61). Our results on the loss of Cavin-2 causing a decrease in the generation of EMPs enhances further understanding of the molecular mechanisms of microparticle formation by lipid microdomain-related proteins and implying a potential role of Cavin-2 in vascular dysfunction.

In summary, we show that Cavin-2 is required for endothelial cell maintenance and function. We further show that Cavin-2, an important component of caveolae, which not only regulates the structural morphology and dynamics of caveolae but also regulates the production of NO in endothelium by activating and stabilizing eNOS (Fig. 8*C*). Also, we find that Cavin-2 is secreted in microparticles and required the generation of microparticles in endothelial cells.

## Experimental procedures

### Zebrafish husbandry, morpholino microinjection, and imaging

Zebrafish (*Danio rerio*) were maintained under standard conditions in the IMCB Zebrafish facility. For studying angiogenesis, we used the *Tg*(*fli1*:EGFP)^y1^ transgenic line (7). All zebrafish procedures in this study are in compliance with the Agri-Food and Veterinary Authority (AVA) of Singapore and approved by the Institutional Animal Care and Use Committee (IACUC) of the Biological Resource Centre (BRC), A*STAR. The eggs were obtained according to protocols described in The Zebrafish Book (63). Morpholinos were injected at a concentration 30 ng/μl into 1-cell stage embryos using a PLI-100 microinjector (Harvard Apparatus). At 24 hpf, fishes were anesthetized with Tricaine (0.02%; buffered to pH 7.0) and mounted in methyl cellulose (3%), and imaged using Olympus confocal microscope.

### OIR on mice

OIR was performed on C57BL/6J mice as previously described (64). Briefly, neonatal mice and nursing mothers were kept at normoxia (normal room atmosphere) until P6 and shifted to 70% continuous oxygen supply (hyperoxia) in a chamber (BioSpherix) with a standard 12-h light-dark cycle from P7 to P12. The higher oxygen content obliterates blood vessel formation and the extent of vaso-obliteration was determined at P12 using retinal flat mounts, see below for more details. The regrowth of blood vessels were induced by keeping mice at normoxia from P12 and the amounts of vessel regrowth were evaluated at P17 using immunofluorescence of α-CD31 and α-Cavin-2 antibodies.

### Retina flat mount and immunofluorescent staining

Mice were euthanized by cervical dislocation and the eyes were immediately enucleated. The eyes were briefly fixed in 4% PFA for 2 min and dissected in 2× PBS. The flattened retinae were then fixed overnight in ice-cold 100% methanol at −20 °C. Retina flat mounts were washed three times in PBS, each for 5 min, before being incubated in blocking solution (consisting 3% Triton X-100, 1% Tween 20, 0.5% bovine serum albumin (BSA) in PBS) for 1 h at room temperature. Retina flat mounts were subjected to overnight incubation at 4 °C in blocking solution containing primary antibodies, α-CD31 and α-Cavin-2. This was followed by incubation with blocking solution containing the corresponding secondary antibodies, goat α-rat IgG-Alexa Fluor 488 and goat α-rabbit IgG-Alexa Fluor 594 (Thermo Fisher Scientific), for 2 h at room temperature. The retinae were then mounted using Mowiol 4–88 mounting medium (Sigma). Using a fluorescence microscope, ×4 objective images were acquired using the Eclipse Ti-E Inverted Research Microscope (Nikon), whereas confocal images were acquired using the Zeiss LSM 800 (Zeiss).

### Expression of Cavin-2 in mouse retina during developmental angiogenesis

C57BL/6 mice were euthanized by cervical dislocation and the eyes were immediately enucleated. For each mouse, the eyes were dissected in 2× PBS and the retina obtained was used for mRNA isolation using RNAzol® RT (Molecular Research Center, Inc.), as per the manufacturer's instructions. Using the C1000 Touch Thermal Cycler (Bio-Rad) and qScript^TM^ cDNA Supermix (Quanta BioSciences), 1 μg of mRNA was reverse transcribed to complementary DNA (cDNA), according to the manufacturer's instructions. The appropriate master mixtures were prepared using PrecisionFAST 2× qPCR Master Mix (with SYBR Green and low ROX) (Primerdesign), qRT-PCR primers, and nuclease-free water (Hyclone) for qRT-PCR and the experiment was conducted using a QuantStudio^TM^ 6 Flex Real-time PCR System (Thermo Fisher Scientific). The expression of Cavin-2 was obtained by normalizing to GAPDH. Nucleotide sequences of primers (mouse) used in qRT-PCR are the following: *Cavin-2* (forward, TTGTGAAGGAGCCAGTTCCC; reverse, TCAGAGGAGAGGTCCACGTT) and *Gapdh* (forward, AGGTCGGTGTGAACGGATTTG; reverse, TGTAGACCATGTAGTTGAGGTCA).

### Cell culture, antibodies, and reagents

HUVEC cells (pooled donor) were purchased from Lonza and cultured in endothelial cell growth medium (Lonza) according to the suppliers instructions. HAEC, HPMEC, and HRMVEC were purchased from Promocell and cultured in endothelial cell growth medium according to the suppliers instructions. For all the experiments, HUVECs and other endothelial cells used were between 2 and 7 passages. The HPMEC-ST1.6R cell line used in this study was a kind gift from R. E. Unger and Prof. C. J. Kirkpatrick at The Institute of Pathology, Johannes Gutenberg-Universität Mainz, Germany. Transfections using siRNAs and plasmids were performed using Lipofectamine RNAiMax and Lipofectamine 2000 (Thermo Fisher Scientific), respectively. The following antibodies were used in this article: α-Cavin-2 (Proteintech), α-phospho-eNOS (Ser-1177), α-phospho-p44/42 MAPK (Erk1/2) antibody (Cell Signaling Technology), α-p44/42 MAPK (Erk1/2) antibody (Cell Signaling Technology), α-eNOS mouse monoclonal (M221) (Abcam), α-β-Actin antibody (Santa Cruz Biotechnology), α-CD31 (WB: Abcam; immunofluorescence: BD Biosciences), α-Caveolin-1 (Abcam), α-GFP (Abcam), α-TSG101 antibody (4A10) (Novus Biologicals), and α,β-Tubulin (Santa Cruz Biotechnology). Also, other reagents include recombinant human VEGF-165 (R&D Biosystems). ON-TARGETplus siRNA reagents for Cavin-2 were purchased from GE Dharmacon. l-NAME and DAF-2 were purchased from Sigma. Cavin-2-mEGFP was a gift from Ari Helenius (Addgene plasmid number 27710) (65).

### Whole cell lysates, immunoprecipitation, and Western blotting

WCL were made by washing cells three times with ice-cold PBS and lysed using modified RIPA lysis buffer (1% Nonidet P-40, 25 mm Tris-HCl, pH 7.4, 150 mm NaCl) with cOmplete protease inhibitor mixture (Roche Applied Science) and PhosSTOP phosphatase inhibitor mixture (Roche). The immunoprecipitations were performed using α-GFP antibodies with WCL made using RIPA buffer (10 mm Tris-HCl, 1 mm EDTA, 1% Nonidet P-40, 0.1% sodium deoxycholate, and 140 mm NaCl). For Western blot, the WCL or immunoprecipitates were boiled for 5 min at 95 °C with Laemmli sample buffer (Bio-Rad), β-mercaptoethanol, and loaded onto SDS-PAGE, transferred to nitrocellulose or PVDF membranes. The Western-blotted membranes were blocked with 5% nonfat dry milk or 5% BSA in Tris-buffered saline with 0.1% Tween 20, immunoblotted with the respective antibodies mentioned in figures, and detected using SuperSignal West Pico Substrate (Pierce) or Luminata Forte Western HRP substrate (Merck). The signals in membranes were exposed in ChemiDoc Touch (Bio-Rad) or X-ray films were scanned and processed using Photoshop CS4 (Adobe) and/or ImageLab 5.2.1 (Bio-Rad).

### Electron microscopy and immunogold labeling

200-Mesh Formvar/carbon nickel grids (Electron Microscopy Services) were glow discharged (Leica) before use. Grids were floated on drops of microparticles for 1 h, before being washed with 0.1 m PBS Grids were fixed using 2.5% paraformaldehyde (Electron Microscopy Services), 0.1% glutaraldehyde (Electron Microscopy Services), 0.2% picric acid (Electron Microscopy Services) in 0.1 m PBS for 1 h before being washed again in 0.1 m PBS. Grids were then probed with α-Cavin-2 antibody at 1:25 in 0.1 m PBS with 0.1% BSA for 1 h at room temperature. Grids were then washed in 0.1 m PBS with 0.1% BSA before being probed with secondary goat anti-rabbit antibody coupled with 10-nm gold (Electron Microscopy Services) for 1 h at room temperature in 0.1 m PBS with 0.1% BSA. Grids were washed in 0.1 m PBS with 0.1% BSA and then 0.1 m PBS. Grids were fixed with 2.5% glutaraldehyde in 0.1 m PBS for 10 min at room temperature before being washed in 0.1 m PBS and distilled water. Grids were stained with 2% uranyl acetate (Electron Microscopy Services), and dissolved in water for 15 min at room temperature before being blotted dry with filter paper. Grids were analyzed by JEM1010 electron microscope (JEOL) operating at 80 kV.

### Cell proliferation and invasion assays

For cell proliferation, around 1000 cells per condition per well were plated in a 96-well plate, grown in EGM-2 media (Lonza). Twenty microliters of CellTiter 96 AQueous One Solution (Promega) were added to each 100 μl of media containing cells and incubated up to 60 min at 37 °C and absorbance was measured at 490 nm using a plate reader (Tecan). For the scratch wound cell migration assay, cells were plated on 12-well plates and allowed to grow to confluence. The confluent cells were scratched using micropipette tips for a regular and even scratch wound and recorded as 0 h using a Nikon cell culture microscope. The cells were allowed to close the wound until 16 h and the results were recorded using a microscope at 16 h. The area of the wound and the cell migration after 16 h were measured using ImageJ. The percentage of migration was calculated based on the relative migration of siCavin-2 with respect to siControl HUVECs. For the transwell migration assay, 1 × 10^5^ HUVECs per condition/well were plated in the upper chamber of 8-μm Transwells and allowed to migrate for 12 h at 37 °C. The migrated cells were fixed with 4% PFA, stained with 0.05% crystal violet (Sigma), washed several times with PBS, and imaged at light microscope (Nikon). The area of the cell migration after 12 h was measured using ImageJ and the percentage of migration was calculated based on the relative migration of siCavin-2 compared with siControl cells. For the cell invasion assay, 8 × 10^4^ cells per well were plated in the upper chamber of a 24-well Biocoat Matrigel invasion chamber (BD Biosciences) and incubated for 48 h at 37 °C. After the incubation, cells in the upper chamber were removed with cotton swabs and cells that invaded the Matrigel to the lower surface of the insert were fixed with 4% PFA and stained with 0. 5% crystal violet, washed several times with PBS, and imaged with a light microscope (Nikon). We have quantified the area of invasion of siControl and siCavin-2 cells using ImageJ and calculated the percentage of relative invasion of siCavin-2 with respect to siControl HUVECs.

### In vitro angiogenesis assays

One hundred microliters of BD Matrigel Basement Membrane Matrix (Matrigel) were loaded in each well of 96-well plates without air bubbles. Matrigel in 96-well plates were allowed to solidify in a 37 °C incubator for 30 min. Endothelial cells were trypsinized and suspended with fresh EGM-2 media. In each well, around 5000–7000 cells in 100 μl of EGM-2 media were carefully loaded on top of the solidified Matrigel and allowed to migrate and form tubular meshwork. The tube formation using endothelia were visualized using a Nikon microscope and the number of branch points and total tube length per well were quantitated using ImageJ.

### Isolation of exosomes, EMPs, and flow cytometry

For the isolation of EMPs, we used a confluent T75 flask of a similar number of HUVECs per condition. Cells were washed three times with Hank's balanced salt solution, the fresh medium was replaced, and the cell culture supernatants were collected after 12 h. The cell culture supernatants were centrifuged for 2000 × *g* for 15 min at 4 °C for removing dead cells and the supernatant was further spun at 10,000 × *g* for 30 min at 4 °C to remove cell debris. The cleared conditioned media (supernatant) were then spun on an ultracentrifuge at 25,000 × *g* for 45 min at 4 °C. The pellets containing EMPs were resuspended in either RIPA buffer (1% Triton X-100, 0.1% sodium deoxycholate, 0.1% SDS, 150 mm NaCl, 1 mm EDTA, 0.5 mm EGTA, and 10 mm Tris-Cl, pH 8.0) with protease and phosphatase inhibitors or in PBS with 0.5 mm EDTA for flow cytometry. For the isolation of exosomes, the supernatants were again centrifuged at 100,000 × *g* for 90 min at 4 °C. The pellets containing exosomes were resuspended in RIPA buffer.

For flow cytometry, isolated EMPs were resuspended in 100 μl of Annexin V binding buffer and stained with Annexin V-FITC as per the manufacturer's protocol (BD Biosciences). For co-staining of Cavin-2 with VE-Cadherin and CD31, we conjugated Cavin-2 antibodies with AMCA using the AMCA conjugation kit (Abcam) and both VE-Cadherin and CD31 with PE with the R-Phycoerythrin conjugation kit (Abcam), as per the manufacturer's protocol. The respective antibodies were incubated with isolated EMPs in the dark at room temperature for 60 min and analyzed using a flow cytometer. For all flow cytometry-based EMP analyses, as an internal reference for size and complexity, nano blank polystyrene size standard kits were used (<1-μm diameters; Spherotech). All measurements were performed with FACS-Canto (BD Biosciences). The results were compared with appropriate isotype controls.

### Isolation of total membrane from endothelial cells

A confluent 10-cm dish of a similar number of HUVEC cells per condition were used for isolation of total membrane. Cells were washed three times with ice-cold PBS and lysed in homogenization buffer (250 mm sucrose, 20 mm HEPES, 10 mm KCl, 1 mm EDTA, 1 mm EGTA, protease and phosphatase inhibitors (Roche Applied Science)) for 15 min on ice. The homogenate were subjected to centrifugation at 3000 rpm for 5 min at 4 °C, then the collected supernatant were centrifuged at 8000 rpm for 5 min at 4 °C. The supernatants were again centrifuged at 40,000 rpm for 1 h at 4 °C. The pellet collected was considered total membrane fractions.

### eNOS activity assay

HUVEC cells were grown in 6-well plates for 24 h. HUVEC cells after knockdown of non-targeting control (siControl) or Cavin-2 (siCavin-2) were growth factor-starved using EGM2 media with 1% fetal bovine serum (FBS) for 12 h, treated with VEGF (50 ng/ml), then incubated at 37 °C and harvested at the mentioned time points. For harvesting, the cells were washed with ice-cold PBS and lysed using lysis buffer as mentioned above.

### Experimental design and statistical rationale

The statistical analyses were performed using Microsoft Excel (Microsoft Inc.) and graphs were made using GraphPad Prism 7 (GraphPad Software Inc.). The dots represent individual data points, middle bars in graphs represent mean values, the top and bottom bars indicate S.D. from three independent experiments (*n* = 3) unless specified. In the article, statistical significance mentioned were determined using paired “two-tailed” Student's *t* tests on the data and are considered significant when a *p* value is <0.05 (represented as *) or represented in respective figure legends of the experiments.

## Author contributions

G. T. K. B. conceived the project, designed, performed experiments, and wrote the manuscript. S. Y. H., M. K., G. T. K. B., and V. A. B. performed mice experiments. G. T. K. B. and T. J. C. performed zebrafish screen. E. W. M. C. performed fluorescence-activated cell sorting of EMPs. X. W. helped with experimental design, expertise, and reagents for mice experiments. W. H. conceived the zebrafish screen and supervised the research.

## Supplementary Material

Supplemental Data

## Footnotes

3 The abbreviations used are:

EGFP: enhanced green fluorescent protein
SDPR: serum deprivation-response protein
eNOS: endothelial nitric-oxide synthase
HUVEC: human umbilical vein endothelial cell
EMP: endothelial microparticles
HPA: Human Protein Atlas
hpf: h post-fertilization
HAEC: human aortic endothelial cell
HPMEC: human pulmonary microvascular endothelial cell
HRMVEC: human retinal microvascular endothelial cells
OIR: oxygen-induced retinopathy
qRT-PCR: quantitative real time PCR
MTT: 3-(4,5-dimethylthiazol-2-yl)-2,5-diphenyltetrazolium bromide
EGM-2: endothelial cell growth medium
DAF-2: 4,5-diaminofluorescein diacetate
l-NAME: *N*^ω^-nitro-l-arginine methyl ester hydrochloride
WCL: whole cell lysate
AMCA: aminomethylcoumarin acetate
PE: R-phycoerythrin
VE: vascular endothelial
PFA: paraformaldehyde.
