# Supplementary material for: Cavin-2 regulates the activity and stability of endothelial nitric-oxide synthase (eNOS) in angiogenesis
Source: J Biol Chem. 2017 Sep 14;292(43):17760–76. doi: 10.1074/jbc.M117.794743 (PMC5663877; doi:10.1074/jbc.M117.794743)
Supplement: Supplemental Data [file supp_292_43_17760__index.html]

Cavin-2 regulates the activity and stability of endothelial nitric oxide synthase (eNOS) in angiogenesis — Cavin-2 regulates the activity and stability of endothelial nitric-oxide synthase (eNOS) in angiogenesis — Cavin-2 regulates nitric oxide levels in endothelial cells — Supplemental Data 

# Cavin-2 regulates the activity and stability of endothelial nitric-oxide synthase (eNOS) in angiogenesis

## Supplemental Data

- Supplemental Figures S1-S4 (.pdf, 694 KB) - Supplemental figures S1, S2, S3 and S4
