# Supplementary material for: Cavin-2 regulates the activity and stability of endothelial nitric-oxide synthase (eNOS) in angiogenesis
Source: J Biol Chem. 2017 Sep 14;292(43):17760–76. doi: 10.1074/jbc.M117.794743 (PMC5663877; doi:10.1074/jbc.M117.794743)
Supplement: Supplemental Data [file 10.1074_M117.794743_jbc.M117.794743-1.pdf]

Supplemental figure S1

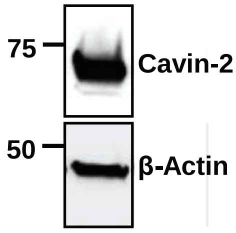

Figure S1. Immunoblot of whole cell lysates (WCL) from human retinal microvascular endothelial cells (HRMVEC) using α-Cavin-2 and α--β-Actin antibodies.

Supplemental figure S2

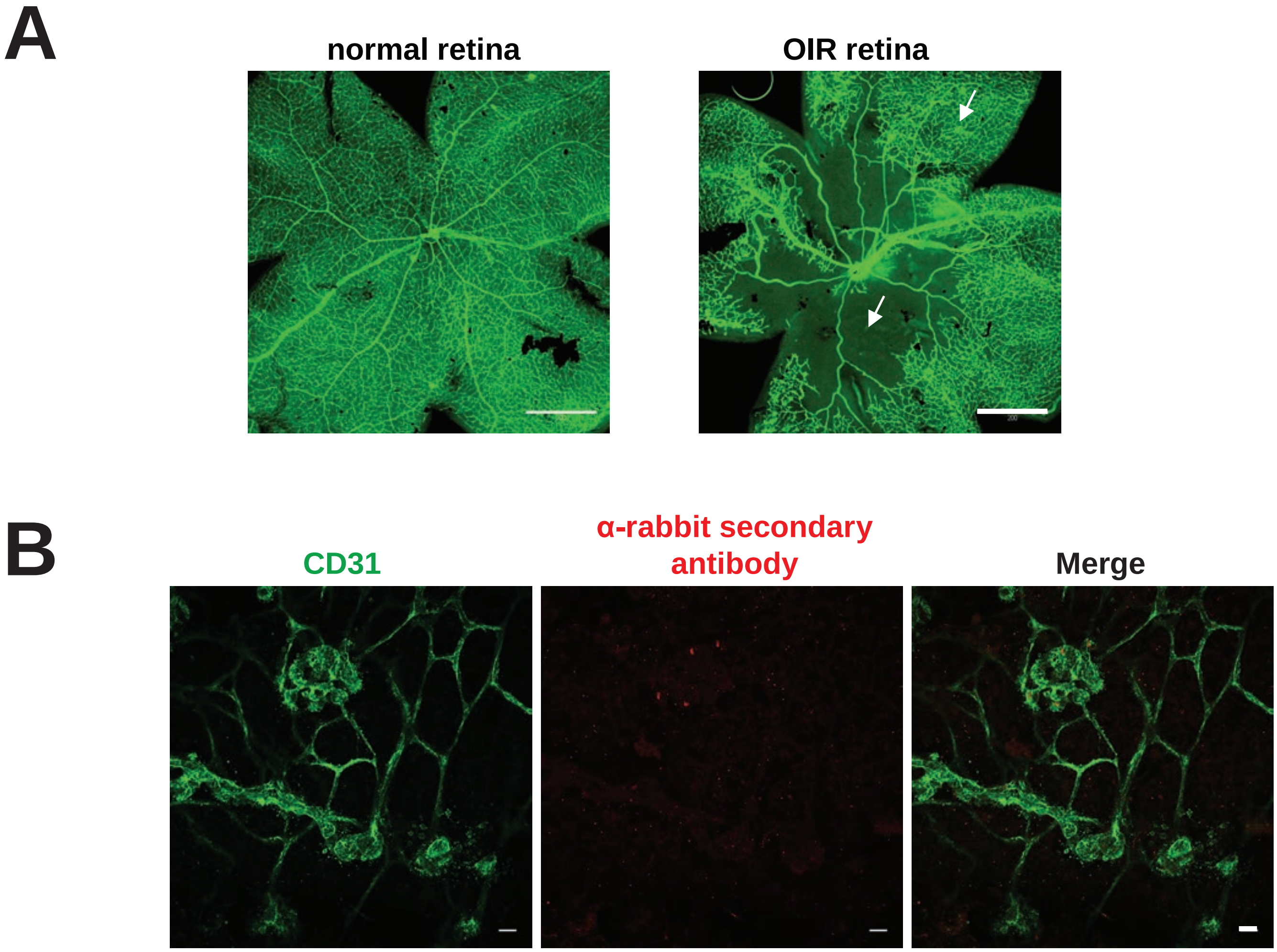

Figure S2. A. CD31 staining in retina of mice maintained in normal oxygen (normal retina) or kept in hyper-oxygen (70%) chamber from P7 to P12 and back to normal room air until P17 (OIR retina). The top and bottom arrows indicate neovascular tufts and the vaso-obliteration (VO) induced by hyper-oxygen in OIR retina, respectively. Scale 200μm B. Immunofluorescence staining of CD31 and α-rabbit secondary antibody on neovascular tufts using OIR in mice.

Supplemental figure S3

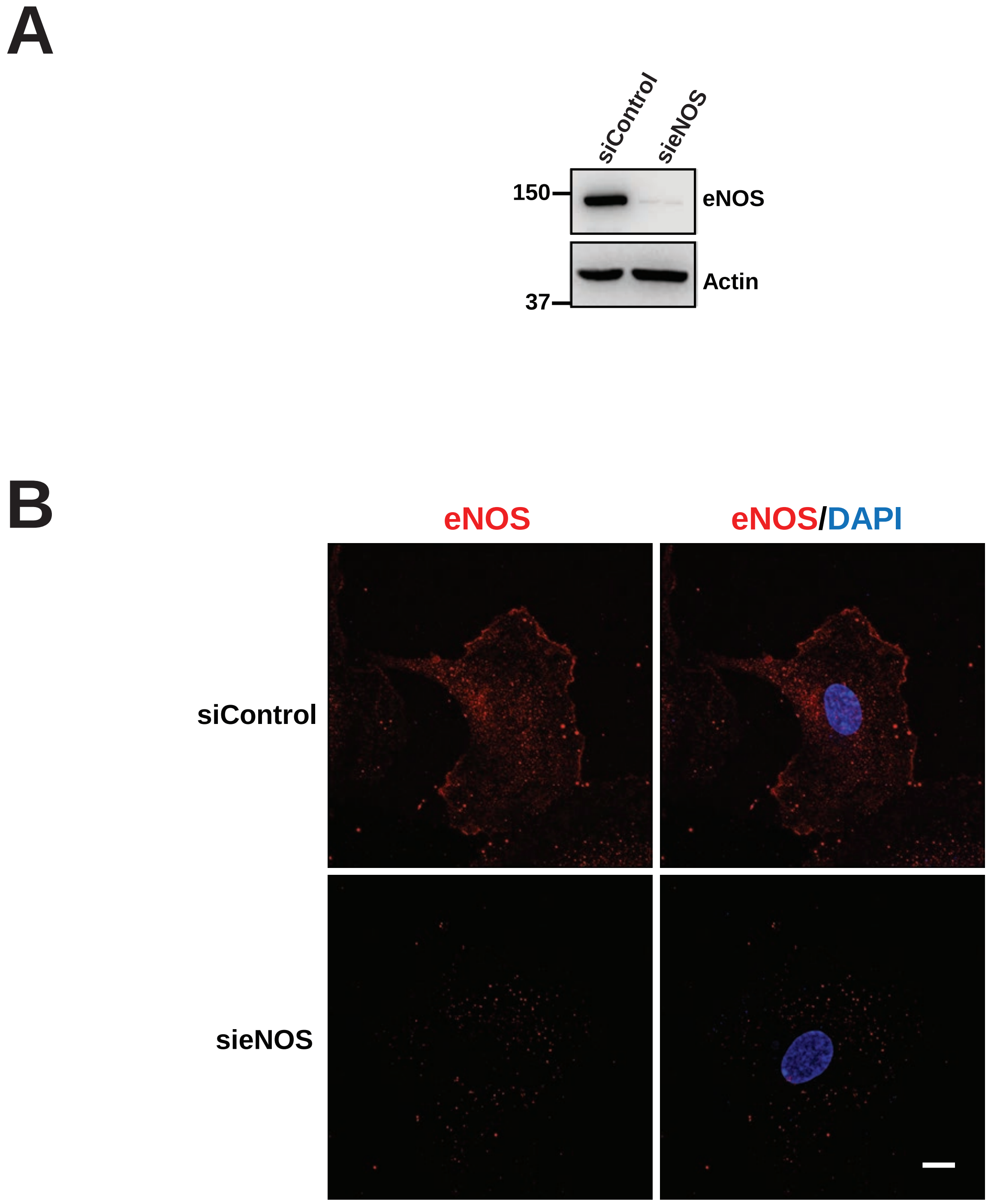

Figure S3. A. Immunoblot of whole cell lysates from HUVECs using eNOS and beta-Actin antibodies after knockdown of non-targeting pool (siControl) and eNOS (sieNOS) siRNAs. B. Immunoflourescence of eNOS on siControl and sieNOS HUVECs. The images are maximum intensity projections recorded using confocal microscope. Scale bar: 10 µm

Supplemental figure S4

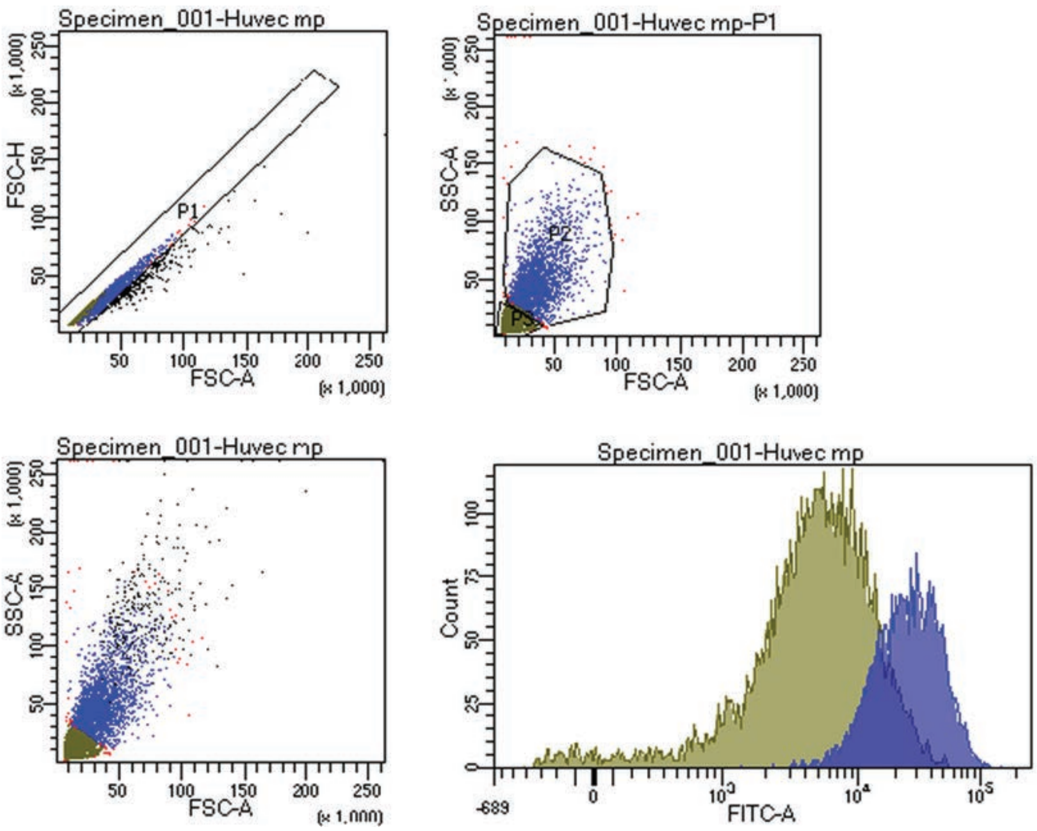

Figure S4. Validation of endothelial microparticles (EMP). EMPs were isolated from HUVECs as mentioned in methods and stained for Annexin-V using Annexin-V-Fluos labelling reagent for one hour at room temperature. The annexin-V stained EMPs were sorted using flow cytometry. The EMPs of size between 0.1-1.0 μm were selected using nano blank polystyrene beads.
